# Cortical iron regulation and inflammatory response in Alzheimer's disease and APP_SWE_/PS1_ΔE9_ mice: a histological perspective

**DOI:** 10.3389/fnins.2015.00255

**Published:** 2015-07-23

**Authors:** Mark D. Meadowcroft, James R. Connor, Qing X. Yang

**Affiliations:** ^1^Department of Neurosurgery, Milton S. Hershey Medical Center, The Pennsylvania State University – College of MedicineHershey, PA, USA; ^2^Department of Radiology (The Center for NMR Research), Milton S. Hershey Medical Center, The Pennsylvania State University – College of MedicineHershey, PA, USA

**Keywords:** Alzheimer's disease, APP/PS1, iron, amyloid-beta plaques, histology, ferritin, microglia, astrocyte

## Abstract

Disruption of iron homeostasis and increased glial response are known to occur in brains afflicted by Alzheimer's disease (AD). While the APP/PS1 transgenic mouse model recapitulates the hallmark amyloid-beta plaque pathology of AD, it does so in a different neuronal mileu than humans. Understanding the iron characteristics and glial response of the APP/PS1 model is important when testing new treatment procedures and translating these results. Brain tissue from AD patients, APP/PS1 mice, and controls were stained for iron, H- and L-ferritin, microglia, astrocytes, Aβ_40∕42_, and degenerating neurons. The histological data demonstrate differences in ferritin, iron distribution, gliosis, and Aβ plaque composition between APP/PS1 and AD tissue. Specifically, an association between focal iron deposition and Aβ plaques is found ubiquitously throughout the AD tissue and is not observed in the APP/PS1 mouse model. Ferritin, microglia, and astrocyte staining show differential response patterns to amyloid plaques in AD and the APP/PS1 tissue. Aβ 40 and 42 antibody and thioflavin staining demonstrate morphological differences in plaque composition. The histological data support the hypothesis that iron distribution, iron management, and glial response histologically differ between the APP/PS1 and AD brain. Acknowledging the caveat that there are distinct plaque, iron, and glial contrasts between the AD brain and the APP/PS1 mouse is crucial when utilizing this model.

## Introduction

The extracellular formation of amyloid-beta (Aβ) protein plaques is a major defining neuropathological characteristic of Alzheimer's disease (AD). It is evident that amyloid formation is involved and relevant in the disease process as plaques are found ubiquitously in AD patients' cortical tissue (Hardy and Selkoe, 2002). Consequently, transgenic mouse models have been developed to mimic the formation of Aβ plaques within neural tissue (Borchelt et al., 1996, 1997) and are especially important for understanding disease etiology and testing new therapeutic procedures (Siman et al., 2000; Jankowsky et al., 2001; Dewachter et al., 2002; Richards et al., 2003; Casas et al., 2004). These specific transgenic mice harbor chimeric mouse/human familial AD genes for amyloid precursor protein (APP) and a mutant human presenilin 1 (PS1) under control of the mouse prion promoter. The mice develop fibrillar Aβ plaques at approximately 9 months of age and subsequently do so progressively throughout their lifespan.

An important histopathologic aspect of Aβ plaques is their co-localization with focal iron deposition (Smith et al., 1996; Lovell et al., 1998). A relationship between iron and amyloid plaques has been established in AD tissue (Smith et al., 1997; Collingwood et al., 2008; Meadowcroft et al., 2009; Ayton et al., 2013) and, to a lesser extent, in the APP/PS1 model (Jack et al., 2004; El Tannir El Tayara et al., 2006; Meadowcroft et al., 2009; Chamberlain et al., 2011; Wengenack et al., 2011; Wadghiri et al., 2012; Bourassa et al., 2013). Iron is an essential element required as a cofactor for numerous metabolic processes due to its ability to receive and donate electrons during redox cycling. Homeostasis of iron is tightly regulated under normal physiological conditions as excessive amounts of iron are known to cause cellular susceptibility to oxidative stress. Accumulation of iron throughout cortical tissue and focal deposition within Aβ plaques are both known to occur within the AD brain (Connor et al., 1992a). In addition, altered regulation of iron management proteins are observed around Aβ plaques and in AD cortical tissue. Specifically, robust staining of intracellular ferritin and extracellular transferrin is observed in the vicinity and periphery of AD Aβ plaques (Connor et al., 1992b). The data strongly suggest that there is a disruption in brain iron homeostasis associated with AD and that the misregulation of iron plays a central role in disease pathology.

Microglial (Benveniste et al., 2001; von Bernhardi and Ramirez, 2001; Lopes et al., 2008) and astrocyte (Schubert et al., 2009) involvement are known to occur in the AD brain and have also been reported in the APP/PS1 mouse brain (Wegiel et al., 2001, 2003). Upregulation of microglia and astrocyte cells are observed in the AD brain and both are involved in iron homeostasis. Microglia participate in neuronal maintenance and protection via the sequestration of excessive iron within ferritin. The incidence of L-ferritin positive active microglia is significantly increased in the AD brain and the characterization of L-ferritin positive cells has shown that they are almost exclusively microglial in nature based on morphological and immunohistochemical (IHC) staining (Jellinger et al., 1990; Connor et al., 1992b; Lopes et al., 2008). An increase in filamentous astrocytes, indicative of activation, is found within AD neural tissue. In addition, astrocyte activation and the astrocyte induced release of reactive oxygen species is mediated by the presence of Aβ in cultured cells (Schubert et al., 2009).

The role of iron misregulation and the ensuing inflammatory response has not been fully elucidated in AD or in the amyloid-generating transgenic mouse models. An animal model that accurately reproduces all characteristics of AD pathology has not been created. Nevertheless, these models are routinely used to study methods of plaque clearance and iron chelation with pharmacological or biochemical interventions (Malm et al., 2012; Guo et al., 2013a; Hajos et al., 2013; Mengel et al., 2013). Investigation of the similarities and differences in the AD and transgenic model brain tissue is essential in understanding how best to use them for AD studies (Woodhouse et al., 2009). With divergent amyloid production processes occurring between the Alzheimer's and APP/PS1 brain tissue, a histological comparison of iron distribution and inflammatory glial response in relation to Aβ plaques within the APP/PS1 transgenic model is important to describe their pathogenic likeness to AD.

In this report, we have undertaken a research design which simultaneously incorporates various histological staining techniques on the same set of tissue samples from the APP/PS1 transgenic mouse model, AD brains, and age-matched controls. Our results provide new information on iron association, inflammatory response, Aβ plaque morphology, and related neurodegeneration in AD and APP/PS1 brain tissue samples which allow us to examine the hypothesis that iron regulation and the associated inflammatory response contrasts between the APP/PS1 and AD brain in these aspects. The data demonstrates that there is a divergence in Aβ plaque iron composition and glial response between the AD and APP/PS1 brain tissue. The rationale for this separation is discussed in regard to brain milieu, glial response, and plaque formation.

## Materials and methods

### Alzheimer's and control brain samples

Entorhinal cortex (Brodmann area 28/34) brain tissue samples from AD subjects (*n* = 5) and age-matched controls (*n* = 3) were obtained with consent and utilized following The Pennsylvania State—College of Medicine Institutional Review Board guidelines (Harvard Brain Tissue Resource Center, McLean Hospital, Belmont, MA) (Demographics in Table 1). Analysis of the tissue indicated that AD tissue samples were highly positive for Aβ plaques and neural fibrillary tangle staining, consistent with a postmortem diagnosis of Braak stage VI (Braak and Braak, 1991; Braak et al., 2006). There was not a significant difference between subjects' age at time of death with Alzheimer's patients averaging 73.6 ± 2.9 years and control patients averaging 75.6 ± 2.9 years. The postmortem interval (PMI) between time of death and tissue harvesting was longer for controls (29.0 ± 1.3 h) compared to Alzheimer's patients (17.3 ± 2.0 h), *p* < 0.01. Tissue dissected from the entorhinal cortex was fully immersion fixed in 4% paraformaldehyde in pH 7.3 phosphate buffered saline (PBS) for 48 h. Tissue samples were cryogenically protected through 10, 20, and 30% sucrose gradients in deionized water (dH_2_O) for 48 h each. Five coronal tissue sections were cut at 16 μm per entorhinal cortex specimen per staining protocol on a cryostat, mounted on poly-lysine and gelatin coated slides, heated to 50°C to adhere samples to slides, and prepared for histological staining according to individual protocols as described below.

**Table 1:** **Summary of Alzheimer's disease and control patient demographics**.

**Patient type**	**Age**	**Sex**	**Braak stage**	**PMI**
Human—Alzheimer's	72	Male	VI	24.13
	75	Male	VI	15.58
	70	Male	VI	11.83
	84	Female	VI	16.83
	67	Male	VI	18.42
Human—Control	70	Male	–	29.36
	80	Female	–	26.67
	77	Male	–	31.16

*PMI, post mortem interval (hours)*.

### Statement of ethical approval

All protocols were approved by The Pennsylvania State University - College of Medicine Institutional Animal Care and Use Committee (IACUC).

### APP/PS1 and control mice

Transgenic mice (*n* = 5) inserted with a chimeric mouse/human APP (APPSwe_695_, K595N and M596L mutations) and a mutant human PS1 (PS1-ΔE9) (Borchelt et al., 1996, 1997) were obtained commercially from The Jackson Laboratory [strain name B6C3-Tg (APPswe,PSEN1dE9)85Dbo/J, stock number 004462]. Animals were kept in the animal facility under veterinary care with normal feeding, light, and handling conditions. All protocols were approved by the Institutional Animal Care and Use Committee. Age-matched non-carrier mice (*n* = 4) were used as controls. After aging naturally until 24 months old, animals were euthanized via an intra-peritoneal injection of sodium pentobarbital (200 mg/kg) and were transcardially perfused with cold Lactated Ringer's solution (pH 7.4), followed by buffered 4% paraformaldehyde in PBS. Whole brain tissue was harvested and placed in pH 7.3 buffered 4% paraformaldehyde for 48 h to allow full tissue fixation. The tissue was cryo-protected in sucrose, cut coronally on a cryostat at approximately Bregma −1.0 mm, and prepared in the same fashion as human sections.

### Iron and amyloid staining

Tissue sections were co-stained for iron and fibrillar Aβ with a 3,3′-diaminobenzidine tetrahydrochloride (DAB) enhanced Perl's Prussian blue stain, followed by an aqueous thioflavin-S stain according to previous methods (Meadowcroft et al., 2009). In brief, mounted tissue sections were rinsed in dH_2_O for 15 min, placed in equal volumes of freshly prepared 4% potassium ferrocyanide (P236, Fisher Scientific, Waltham, MA) and 4% hydrochloric acid (HCl) (final combined concentrations 2% for each) for 30 min, followed by two 5 min rinses in dH_2_O. Intensification of the iron stain was performed with 5 min of DAB counterstaining (D5637, Sigma, St. Louis, MO) (10 mg dissolved in 15 ml of PBS with 16 μl of 30% H_2_O_2_) followed by two 5 min rinses in dH_2_O. Tissue samples were then placed in 1% thioflavin-S (T1892, Sigma, St. Louis, MO) aqueous solution for 5 min, followed by differentiation in 70% ethanol for 5 min and two 5-min washes in dH_2_O. To preserve fluorescence, sections were covered with aqueous mounting media and cover slipped. To test for the possibility of confounding interactions between methods during co-staining; each stain was tested on separate tissue samples to determine their individual efficacy compared to the co-stained sections. The results of this test indicated no adverse interaction between selected stain pairings. In addition, tissue sections placed in DAB alone demonstrated there was no binding of the compound to Aβ plaques. A modified Perl's stain (LeVine, 1991, 1997) with proteinase K was used to visualize minute amounts of iron within the amyloid masses. Tissue sections were hydrated in PBS for 15 min followed by immersion in sodium borohydride (10 mg/ml PBS, 213462, Sigma, St. Louis, MO) for 30 min. Sections were then rinsed in PBS twice for 5 min and immersed in proteinase K (30 μg/ml, P6556, Sigma, St. Louis, MO) and 0.01% Triton X-100 in PBS for 20 min. Next, the sections were placed in a solution of 1% hydrochloric acid, 1% potassium ferrocyanide, and 1% Triton X-100 in distilled water for 30 min. Amplification of the iron staining was accomplished with 0.5 mg/ml DAB and 2 μl/ml of 30% hydrogen peroxide in pH 7.6 0.05 M Tris HCl for 15 min. The sections were rinsed twice for 5 min in dH_2_O, stained with thioflavin-S aqueous solution for 5 min, differentiated in 70% ethanol for 5 min, and finally underwent two 5 min dH_2_O rinses before being mounted on slides and cover slipped.

### Immunohistochemistry staining

Air dried tissue sections were placed in 95°C citrate buffer (10 mM sodium citrate tribasic dehydrate, 0.05% Tween 20 in dH_2_O, pH 6.0) for 15 min. Slides were rinsed three times with PBS for 5 min each, then nonspecific protein binding was blocked by 30 min incubation with 1% bovine serum albumin (BSA) in 1x PBS Tween (PBST). Sections were incubated with primary antibodies at their respective dilution factors in 1% BSA in 1x PBST overnight in an airtight incubation-humidity chamber. After primary incubation, the antibody was decanted and slides were rinsed in PBS three times for 5 min each. Sections were incubated with fluorescent secondary antibodies (Alexa Fluor, Invitrogen, Carlsbad, CA) in 1% BSA in 1x PBST for 2 h in a humidity chamber. The secondary antibody was then decanted and slides were rinsed in PBS three times for 5 min each, followed by mounting, and cover-slipping.

For Aβ_40_ staining, a mouse monoclonal beta Amyloid 1-40 antibody [BAM-10] (1/100, AB7501, Abcam Inc., Cambridge, MA) was used followed by an anti-mouse red fluorescent Alexa Fluor 555 IgG (A-21422) secondary antibody. Following secondary incubation and PBS rinsing, tissue sections were co-stained with 1% thioflavin-S for 10 min, differentiated in 70% ethanol, washed twice in dH_2_O, mounted, and cover-slipped.

For Aβ_42_ staining, a rabbit polyclonal beta Amyloid 1-42 antibody (1/200, AB10148, Abcam Inc., Cambridge, MA) was used followed by an anti-rabbit red fluorescent Alexa Fluor 555 IgG (A-21428) secondary antibody. Tissue sections were then co-stained with thioflavin-S according to the method outlined above.

To stain for light-ferritin (L-) polypeptides, a mouse monoclonal ferritin light chain (D-9) antibody (1/125, SC-74513, Santa Cruz Biotechnology, Santa Cruz, CA) was used followed by Alexa Fluor 555 IgG (A-21422) secondary antibody. Heavy-Ferritin (H-) polypeptides were stained with a rabbit polyclonal ferritin heavy chain (H-53) antibody (1/125, SC-25617, Santa Cruz Biotechnology, Santa Cruz, CA), followed by Alexa Fluor 555 IgG (A-21428) secondary antibody. Tissue sections were co-stained with thioflavin-S to stain for fibrillar Aβ deposits.

To stain for microglial cells, a rabbit Anti IBA-1 (Ionized calcium binding adaptor molecule 1) antibody (1/500, 019-19741, Wako Chemicals USA, Inc., Richmond, VA) was used, followed by incubation with Alexa Fluor 555 IgG (A-21428) secondary antibody. Thioflavin-S was used as a co-stain for fibrillar Aβ after antibody staining.

To distinguish astrocytes, a polyclonal chicken anti-glial fibrillary acidic protein (GFAP) antibody (1/250, AB5541, Millipore, Billerica, MA) was used, followed by Alexa Fluor 555 anti-chicken IgG (A-21437) secondary antibody incubation. Tissue sections were then stained with thioflavin-S for fibrillar Aβ visualization.

### Fluoro-Jade C staining

A Fluoro-Jade C (FJC) stain was utilized along with immunohistological antibody staining to identify degenerating neuronal cells and their relation to Aβ plaques. Sections were stained with Aβ_40_ specific primary antibodies, followed by Alexa Fluor 555 secondary antibody according to the procedure above and then rinsed twice for 5 min in PBS. Slides were then rinsed in dH_2_O for 2 min followed by immersion in 0.06% potassium permanganate for 10 min and a subsequent rinse for 2 min in distilled water. Slides were immersed in 0.0001% FJC (AG325, Millipore, Billerica, MA) solution for 10 min, followed by three 1 min rinses in dH_2_O. Slides were then air dried for 30 min at 50°C followed by immersion in xylene dehydration, mounted, and cover-slipped. The exact mechanism by which Fluoro-Jade works has not been resolved, however it is hypothesized that the highly poly-anionic fluorescein derivative binds to a specific apoptotic molecule which is expressed by damaged neuronal cells (Schmued et al., 2005).

### Microscopy

High resolution microscopy of cortical gray matter regions of interest in the tissue sections was performed using a Nikon OptiPhot microscope and Nikon Digital Sight camera using NIS-Elements software. Bright-field under the visible light spectra and phase contrast using a phase contrast objective and condenser were used to view iron stains. A FITC fluorescence cube at 495 nm excitation and 520 nm emission (Nikon B-22) was used to visualize thioflavin-S positive Aβ deposits and FJC stains. A Nikon G-2A filter cube at 550 nm excitation and at 570 nm emission was used to visualize the Alexa Fluor 555 secondary antibody.

## Results

**Figure 1:**
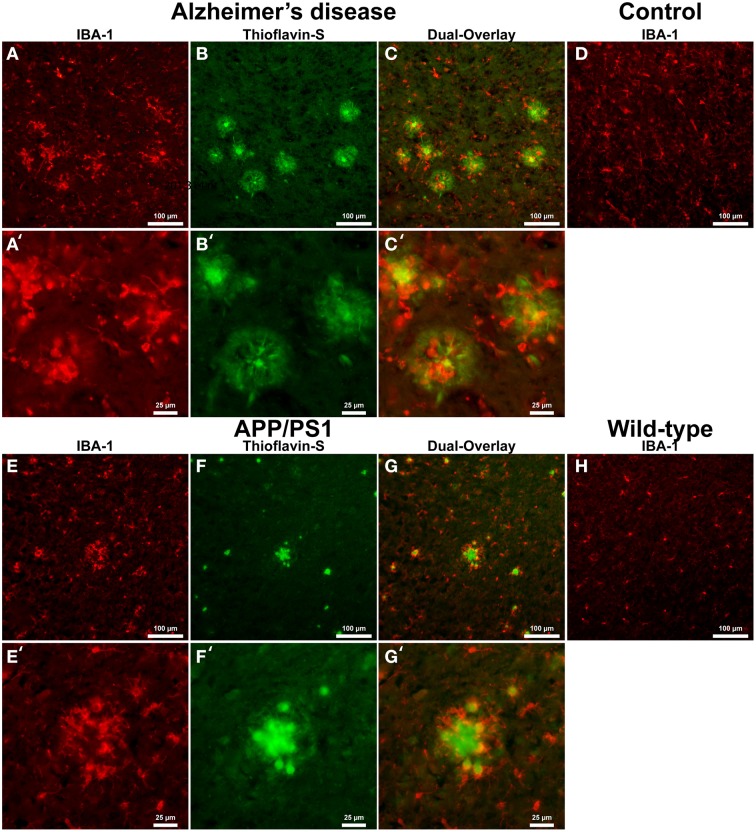
**Ionized calcium-binding adaptor molecule - 1 (IBA-1) antibody staining for microglia (red, A,E) and thioflavin-S (green, B,F) for amyloid-β plaques in Alzheimer's disease (A–C), APP/PS1 (E–G), control human (D) and control mouse (H) tissue samples at 100x (A–H) and 400x (A′–C′,E′–G′) magnification**. Microglia in the AD samples are associated with amyloid-β plaque location and are seen in an activated amoeboid state surrounding plaques with processes infiltrating into the coronal region. In the APP/PS1 tissue, microglial cells are also observed in an activated state surrounding Aβ plaques. Human control tissue samples show positive microglial staining and demonstrate microglial cells in an intermediate ramified resting state with numerous processes surrounding their somas. Mouse control tissue exhibits less microglial staining overall and shows cells in a quiescent resting state. The data demonstrates a subtle difference in microglial inflammatory response between AD and APP/PS1 neural tissue samples. Scale bars are 100 and 25 μm in length.

IBA-1 positive microglial cells in human AD (Figure 1A) show microglia morphologically in both ramified and active states throughout the tissue samples. Thioflavin-s staining (Figure 1B) of Aβ plaques illustrates a close proximal distribution of microglia around the periphery and within AD Aβ plaques (Figure 1C). Higher magnification of AD sections details the association with Aβ plaques and activated microglia (Figures 1A′–C′). Microglial bodies and processes are found both surrounding and within the fibrillar corona region of the AD plaques. Age-matched human control tissue (Figure 1D) demonstrates IBA-1 positive microglial cells throughout the cortical tissue in both ramified and active states. Human control tissue does not exhibit microglial clustering as seen in the AD tissue. The total amount of microglial staining in human control tissue visually appears similar to AD tissue. Transgenic APP/PS1 tissue exhibits both activated and ramified microglia based on cellular morphology (Figure 1E). An association between microglia and the periphery of Aβ plaques is observed with activated microglia surrounding transgenic plaques (Figures 1E–G, and magnification Figures 1E′–G′). Mouse control tissue (Figure 1H) demonstrates sporadic ramified microglial staining. There qualitatively appears to be similar microglia staining in the mouse control tissue compared to that of APP/PS1 tissue samples. Human and mouse control tissue did not stain positive for thioflavin-S plaque formations (not shown).

**Figure 2:**
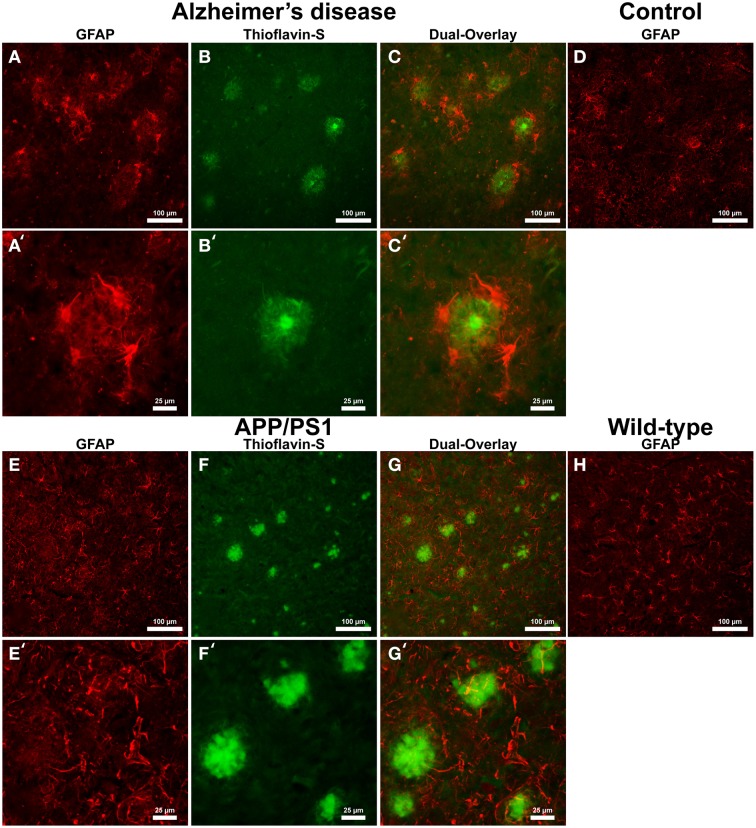
**Glial fibrillary acidic protein (GFAP) (red, A,E) for astrocytes and thioflavin-S (green, B,F) staining for Aβ plaques in Alzheimer's disease (A–C), APP/PS1 (E–G), control human (D) and control mouse (H) tissue at 100x (A–H) and 400x (A′–C′,E′–G′) magnification**. Astrocytes in the AD tissue are in a motile phagocytic state and surround the coronal region of the Aβ plaques. In human control tissue, the astrocytes are observed in their normal highly branched form indicative of their supportive roll for neuronal tissue. APP/PS1 and mouse control tissue similarly stain positive for GFAP throughout the tissue samples and illustrate astrocytes in a highly branched normal supportive role. The strong association seen between astrocytes and Aβ plaques in the AD tissue is not seen in the transgenic model. The data indicates a differential astrocytic inflammatory response to Aβ plaques in the natural AD milieu compared to the transgenic model. The scale bars are standardized to 100 and 25 μm.

Astrocyte IHC staining in AD tissue (Figure 2A) samples illustrate GFAP positive cells throughout the microscopic field and around the periphery of Aβ plaques (Figures 2B,C). Astrocytes surround Aβ plaques rather than displaying a random distribution throughout the cortex. Magnification of the AD tissue demonstrates astrocyte dense arm morphology indicative of an activated phagocytic astrocyte state (Figures 2A′–C′) (Schubert et al., 2009). Astrocytic processes are radially infiltrating into the halo region toward the core of the AD Aβ plaques. Human control tissue (Figure 2D) illustrates astrocytes in a traditional star pattern, with radiating processes typical of astrocytes acting as neuronal metabolic helper cells. Transgenic APP/PS1 mouse tissue sections exhibit an indiscriminate distribution of GFAP positive astrocytes throughout the microscopic field (Figures 2E–G). Magnification of Aβ plaques in the APP/PS1 tissue demonstrates they are not surrounded by GFAP positive astrocytes, but show a random staining pattern (Figures 2E′–G′), converse to Alzheimer's samples. Mouse control tissue displays a similar GFAP positive astrocyte distribution and reactive state to APP/PS1 tissue (Figure 2H).

**Figure 3:**
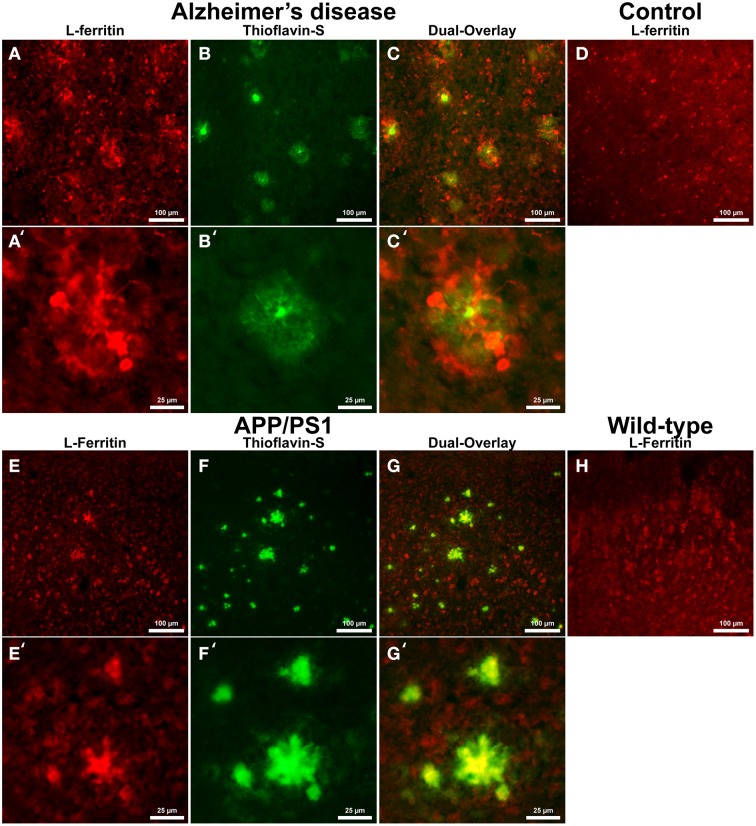
**Light ferritin antibody (red, A,E) and thioflavin-S (green, B,F) staining for amyloid-β plaques in AD (A–C), APP/PS1 (E–G), control human (D) and control mouse (H) tissue at 100x (A–H) and 400x (A′–C′,E′–G′) magnification**. L-ferritin staining in the AD tissue samples shows intracellular staining in cells surrounding amyloid plaques and throughout the imaging field. The cellular morphology of the L-Ferritin positive cells in the AD tissue is identical to the cells stained for IBA-1 (see Figure 1 for comparison). Human control tissue **(D)** L-ferritin reactivity is found in small round oligodendrocyte cells throughout the cortex with some microglial staining consistent with aged human gray matter. L-ferritin staining in the APP/PS1 tissue shows intracellular staining of cells inside plaques and oligodendrocytes in the granular layer of the cortex which. In contrast to the AD tissue, there is no positive staining of cells surrounding amyloid plaques in the APP/PS1 tissue samples. Mouse control tissue **(H)** demonstrates intracellular L-ferritin reactivity in oligodendrocyte cells, similar to the background oligodendrocyte staining in the APP/PS1 tissue. The scale bars are set to 100 and 25 μm in length.

**Figure 4:**
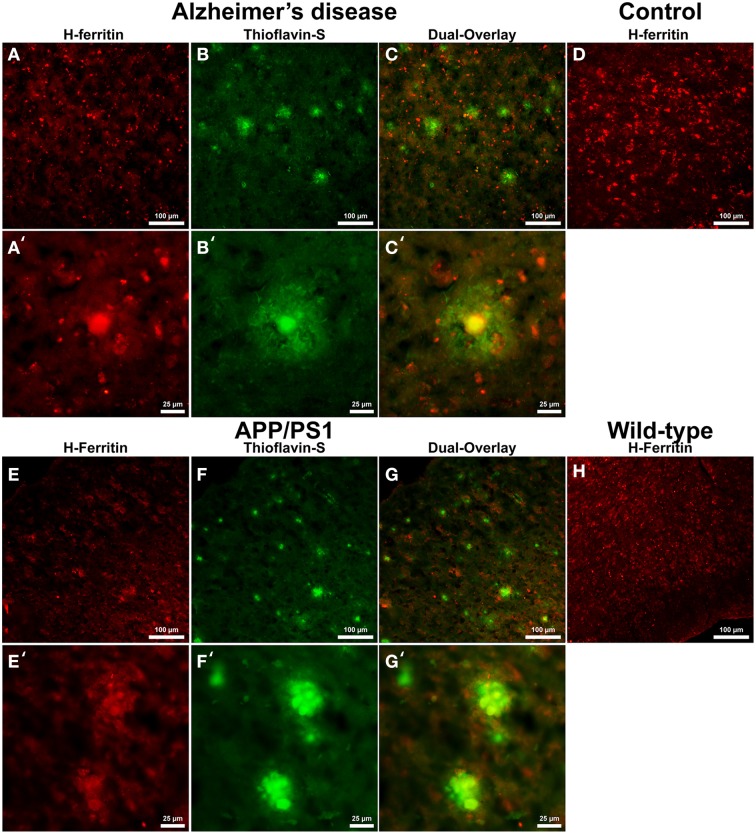
**Heavy ferritin antibody (red, A,E) and thioflavin-S (green, B,F) staining for amyloid-β plaques in AD (A–C), APP/PS1 (E–G), control human (D) and control mouse (H) tissue at 100x (A–H) and 400x (A′–C′,E′–G′) magnification**. H-ferritin staining of AD samples demonstrates positive intracellular neuronal staining throughout the tissue samples in relation to amyloid-β plaque core location. Human control tissue **(D)** demonstrates positive H-ferritin staining in neurons throughout the microscopic field. There was slight positive intraneuronal staining for H-ferritin in the APP/PS1 tissue samples. H-ferritin positive immunoreactivity was found in the core of APP/PS1 Aβ plaques. Mouse control tissue stained positive for intraneuronal H-ferritin throughout the cortical tissue. Scale bars are 100 and 25 μm in length.

Cells positive for light (L-) ferritin staining in AD tissue (Figure 3A) are found in close proximity to thioflavin-S positive Aβ plaques (Figures 3B,C) and throughout the cortical gray matter field of view. The morphology of numerous L-ferritin stained cells associated with Aβ plaques are similar to IBA-1 positive cells visualized in Figure 1, highlighting intracellular L-ferritin within microglial cells. Closer magnification shows that L-ferritin positive cells found within the coronal region of the Aβ plaques are microglia (Figures 3A′–C′) based on cellular morphology and prior research demonstrating L-ferritin accumulation predominantly in AD microglia (Kaneko et al., 1989; Grundke-Iqbal et al., 1990). Tri-staining of L-ferritin, thioflavin-S, and IBA1 was not possible due to overlapping fluorescent emission spectra of secondary antibodies and thioflavin. L-ferritin immunoreaction visualized in numerous small round cells dispersed throughout layers of the entorhinal cortex are consistent with perivascular oligodendrocyte morphology (Connor and Fine, 1986, 1987; Connor et al., 1990). Human control tissue (Figure 3D) L-ferritin immunoreactivity is primarily found in oligodendrocytes with some microglial staining in a pattern consistent with aged human gray matter (Connor et al., 1990). Positive L-ferritin reactivity within the APP/PS1 tissue (Figure 3E) is found in cells throughout the cortex and in relation to Aβ plaques (Figures 3F,G). Small round cells positive for L-ferritin reflect previously described oligodendrocyte morphology in both the transgenic APP/PS1 (Figure 3E) and control tissue (Figure 3H). Higher magnification demonstrates L-ferritin reactive cells within the core of Aβ plaques (Figures 3E′–G′). While AD tissue shows a close morphological overlap of microglia with IBA-1 and L-ferritin staining, it is not readily apparent if the cells found within the APP/PS1 Aβ plaques are microglia. Transgenic cells positive for L-ferritin outside of the plaques appear within the granular layer of the cortex and morphologically appear to be oligodendrocytes. A similar distribution of L-ferritin immunoreactivity is visualized in the mouse control tissue within oligodendrocyte cells (Figure 3H). Intracellular heavy (H-) ferritin immunoreactivity, which appears neuronal in origin, is found in cells throughout the AD cortex (Figures 4A–C) (Thompson et al., 2003). Magnification of Aβ plaques (Figures 4A′–C′) illustrates positive H-ferritin staining within the plaque core which is absent from the halo region. Human control tissue exhibited positive H-ferritin staining of neuronal cells throughout the microscopic field of view (Figure 4D). Intraneuronal staining for H-ferritin was viewed in the APP/PS1 transgenic animals (Figure 4E) to a lesser degree than AD samples. The relationship between H-ferritin and the APP/PS1 Aβ plaques (Figures 4F,G) is realized at higher magnification (Figures 4E′–G′), at which H-ferritin positive immunoreactivity is found in the Aβ plaque core. Mouse control tissue (Figure 4H) stained positive for intraneuronal H-ferritin immunoreactivity throughout the microscopic field.

**Figure 5:**
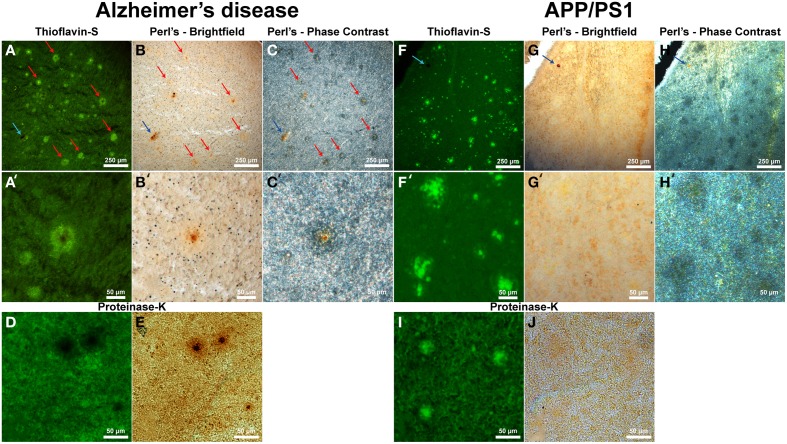
**Alzheimer's disease (A–E) and APP/PS1 (F–J) tissue stained with thioflavin-S and a traditional Perl's stain under fluorescence (left), bright-field (middle), and phase contrast (right) microscopy at 40x (A–J) and 200x (A′–H′) magnification**. Red arrows indicate selected plaques that have ferric iron associated with them, while the blue arrow indicates a focal iron region not associated with an Aβ plaque and is composed of hemosiderin or magnetite. The core of numerous dense core Aβ plaques in the AD tissue samples exhibit focal iron deposition and diffuse iron throughout their coronal regions in both the phase contrast and bright-field image sets. The APP/PS1 transgenic mouse tissue does not stain positive for focal iron associated with fibrillar Aβ plaques with the Perl's stain. The blue arrow illustrates a focal iron deposit not associated with a plaque that indicates the Perl's stain is effective in staining ferric iron deposits. Phase contrast microscopy shows regions where fibrillar thioflavin-s positive Aβ plaques are located, as well as thioflavin-s negative regions beyond the fibrillar deposition. The modified thioflavin-S and Perl's stain use protein digestion to allow further penetration of the aqueous stain into the hydrophobic AD **(D,E)** and APP/PS1 **(I,J)** plaques. The stain indicates that there is a very minute amount of iron found in the APP/PS1 plaques **(J)** that is not stainable with traditional Perl's staining methodology. Human plaques no longer stain positive for thioflavin-S **(D)** due to Aβ protein degradation and subsequent lack of thioflavin-S Aβ fibril intercalation, however the morphology of the ferric iron staining verifies plaque location. There is a large visual amount of iron in the core and halo region of the AD tissue **(E)** that is not seen in the transgenic tissue. Scale bars are 250 and 50 μm.

Thioflavin-S staining (Figure 5A and higher magnification in Figure 5A′) shows numerous dense core plaques throughout the AD samples that stain positive for focal iron deposition (red arrows). These iron stains can be seen in both the phase contrast (Figures 5C,C′) and bright-field (Figures 5B,B′) images. The phase contrast image shows the outline of the Aβ plaques as opaque due to the light wave phase shift when passing through the plaques. The APP/PS1 tissue exhibits positive thioflavin-S staining for Aβ plaques (Figures 5F,F′), but does not exhibit focal iron staining in either the phase contrast (Figures 5H,H′) or the bright-field images (Figures 5G,G′). Positive iron staining was found sporadically within the transgenic tissue, but did not co-register to Aβ plaques (blue arrow) indicating positive Perl's-DAB staining. Similar to the human AD data, the APP/PS1 plaques also exhibit a difference in light passage in the phase contrast images. The phase contrast for the Alzheimer's data shows a close approximation of plaque size compared to the thioflavin-S staining. Phase contrast images of the transgenic mouse tissue demonstrate that Aβ plaques are larger in diameter than when visualized with the thioflavin-S alone.

While the traditional Perl's stain shows a lack of detectable ferric iron in the APP/PS1 plaques, there is a minute amount of iron associated with the transgenic Aβ plaques when stained with a modified Perl's stain and compared to the Alzheimer's plaques (Figures 5D,E,I,J). The modified iron stain incorporates protein digestion of Aβ fibrils to enable the aqueous Perl's iron stain further access into the AD and APP/PS1 plaques. Treatment with proteinase K results in the digestion of AD Aβ plaques causing thioflavin-S negative staining for these plaques. This staining result is presumably due to the degradation of the component Aβ fibrils resulting in an inability to intercalate the thioflavin-S molecule(s). The modified Perl's stain indicates the locations of AD Aβ plaques based on iron staining morphology; the core of the AD plaques is high in focal iron with less staining in the coronal regions. The periphery of APP/PS1 plaques is moderately digested, allowing thioflavin-S binding, illustrating a difference in the ability of proteinase K to break down the transgenic plaques. We hypothesize that this contrast is caused by the denser Aβ fibril core composition of the transgenic plaques compared to the AD plaques (Meadowcroft et al., 2009).

**Figure 6:**
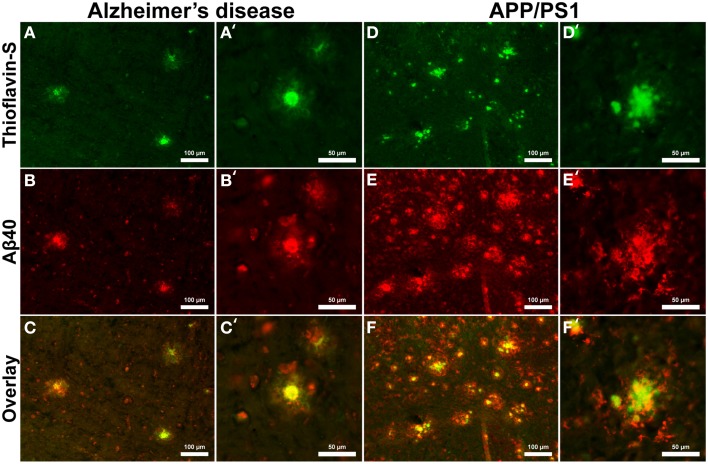
**Antibody stains for Aβ_40_ (red) and thioflavin-S (green) of Alzheimer's disease (A–C) and APP/PS1 (D–F) cortical tissue viewed at 40x (A–F) and 100x (A′–F′) magnification**. Aβ_40_ is found within plaques in the AD and transgenic tissue samples as well as intracellular staining throughout both samples. Thioflavin-S staining of fibrillar Aβ demonstrates that the core of the AD plaques is composed of highly fibrillar filaments. Antibody staining illustrates that the plaque core contains Aβ_40_ with less staining in the coronal region. The APP/PS1 tissue exhibits increased Aβ_40_ staining, indicating over production of the 40 amino-acid constituent. APP/PS1 plaques also display different morphology with a globular fibrillar structure that radially extends from the center of plaque. Antibody staining with Aβ_40_ illustrates that the size of the APP/PS1 plaques is underestimated with the fibrillar thioflavin-S stain alone as the antibody stains both fibrillar and protofibril Aβ, demonstrating that the periphery of the transgenic plaques is composed of protofibril filaments. This overestimation is similar to the phase contrast images of APP/PS1 plaques in Figure 5. Scale bars are 100 and 50 μm.

**Figure 7:**
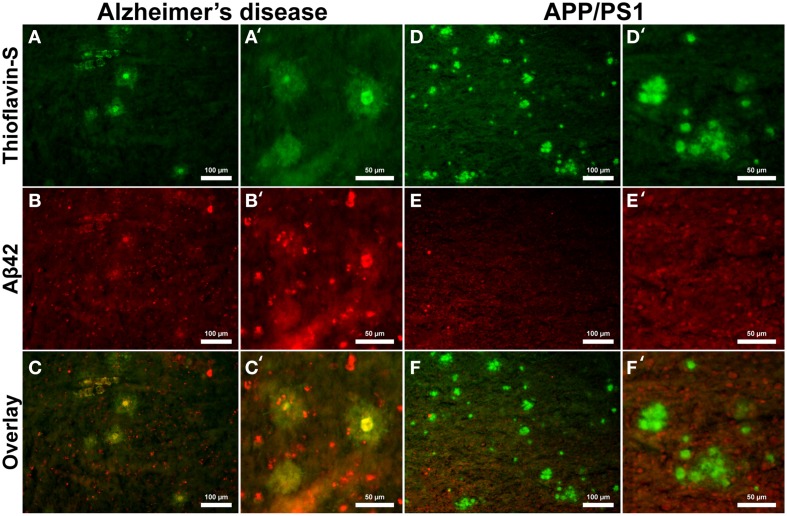
**Antibody stains for Aβ_42_ (red) and thioflavin-S (green) of Alzheimer's disease (A–C) and APP/PS1 (D–F) cortical tissue viewed at 40x (A–F) and 100x (A′–F′) magnification**. Alzheimer's samples show positive Aβ_42_ staining in the core and coronal regions of the plaques as well as intracellularly throughout the microscopic field. The antibody stains indicate that the core and coronal regions of the Alzheimer's plaques are composed of both 40 and 42 amino-acid variants; see Figure 6 for comparison. The APP/PS1 tissue stained minimally for intracellular Aβ_42_, which was not associated with transgenic plaques. The Aβ stains indicate that APP/PS1 plaques are composed primarily of the 40 amino-acid constituent. Scale bar is calibrated to 100 and 50 μm.

Aβ_40_ composition of plaques in relation to fibrillar thioflavin-S stains are markedly different in AD than in APP/PS1 tissue samples (Figure 6), with clear contrast in plaque morphology. Thioflavin-S binding of AD plaques (Figures 6A,A′) shows a dense fibrillar core surrounded by a large diffuse coronal halo region. APP/PS1 thioflavin positive plaques (Figures 6D,D′) exhibit a larger dense core region with a smaller diffuse thioflavin-S positive coronal region. Fluorescent conjugated Aβ_40_ antibodies bound to AD plaques (Figures 6B,B′) stain both the dense core and coronal regions for the 40 amino acid Aβ variant, with a clear overlap of thioflavin-S staining (Figures 6C,C′). APP/PS1 plaques exhibit Aβ_40_ protofibril reactivity (Figures 6E,E′) that extends beyond the thioflavin positive boundary of the large core and coronal regions (Figures 6F,F′). This is similar to the results in Figure 5 where phase contrast imaging of APP/PS1 tissue reveals diffuse amyloid larger in diameter then the fibrillar thioflavin-S stain. Human AD and APP/PS1 tissues both show positive intracellular Aβ_40_ reactivity in numerous cells outside of the plaques. Antibody staining with Aβ_42_ (Figures 7B,B′) shows that much of the dense fibrillar core of the human plaques (Figures 7A,A′) is composed of the 42 amino-acid Aβvariant, with some staining in the coronal region (Figures 7C,C′ overlay). APP/PS1 tissue did not stain inside or around the beta-amyloid plaques for Aβ_42_ (Figures 7D–F, D–F′). Apparent intracellular staining of Aβ_42_ is found in numerous human AD cells throughout the imaging field and is minimally found within the transgenic tissue samples. It is important to note that the Aβ plaques in the transgenic model are also recognized by human antibodies, as the transcribed peptides are derived from human mutations (Schwab et al., 2004).

**Figure 8:**
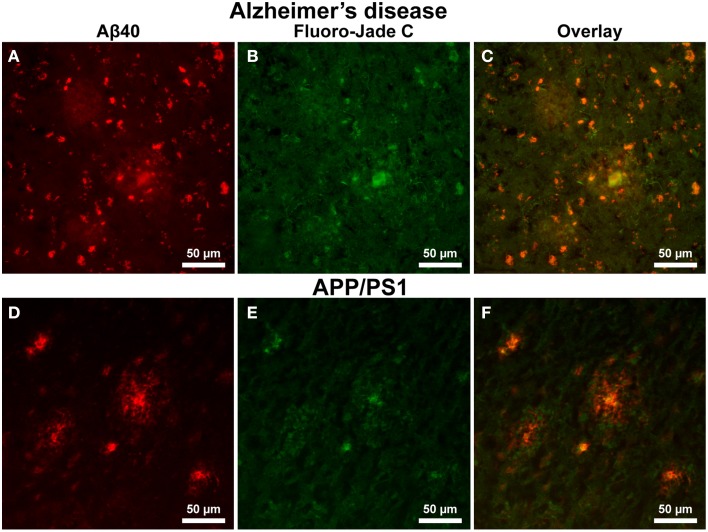
**Amyloid-β 40 staining (red) and Fluoro Jade-C (green) staining in Alzheimer's and APP/PS1 tissue sections at 200x magnification**. Aβ plaque staining is similar to that seen in Figure 6 for both AD **(A–C)** and APP/PS1 **(D–F)** tissue samples with positive plaques and intracellular Aβ_40_ staining. Fluoro Jade-C staining is positive for degenerating neurons in the AD tissue sample and overlaps with intracellular Aβ_40_ staining. The APP/PS1 tissue demonstrates fluoro Jade-C positive staining within the core of the transgenic plaques and a lack of staining for degenerating neurons surrounding plaques. The scale bar is 50 μm in length.

Alzheimer's tissue exhibit positive FJC staining in both the interior core of Aβ plaques and within cells outside of the coronal halo regions (Figures 8A–C, 200x magnification). Additionally, a precise overlap exists between FJC and Aβ_40_ staining in numerous cells within the AD tissue (Figure 8A). In contrast, positive FJC staining in APP/PS1 mouse tissue (Figures 8D–F) samples is observed in the central core of Aβ plaques, while no FJC or Aβ_40_ staining is seen in cells surrounding amyloid plaques.

## Discussion

While regulation of iron has been shown to be associated with the neurodegenerative processes in AD (Gerlach et al., 1994), the causative relationship between disease pathology and iron regulation has not been resolved. Previous work has determined that metal ions play a role in Aβ fibril formation; however, it is unclear how Aβ plaque aggregation is initiated. Aβ has been characterized as a metalloprotein that binds Fe^3+^ and the incorporation of the Aβ fibrils into plaque assemblies is accelerated in an iron-enriched environment (Bush, 2002). Iron-laden Aβ plaques are major sites for catalytic redox activity, especially when combined with high concentrations of iron (Huang et al., 1999; Sayre et al., 2000; Smith et al., 2000; Maynard et al., 2005; Harman, 2006; Khan et al., 2006). Co-localization of iron within Aβ plaques is accompanied by endoplasmic reticulum stress induced apoptosis, DNA oxidation, and cellular damage in cells adjacent to plaques (Atwood et al., 1999; Perry et al., 2002; Lovell and Markesbery, 2007). Additionally, in regions where Aβ plaques accumulate without the marked presence of iron there is no indication of oxidative stress or apoptotic activation (Ghribi et al., 2006); this contrast strongly suggests that iron accumulation in and around plaques promotes cellular damage. Furthermore, studies have demonstrated that the toxicity of the Aβ peptide is amplified upon the direct interaction of iron ions (Rottkamp et al., 2001). The escalated presence of uncomplexed iron in the Alzheimer's brain increases the likelihood of an amyloid-iron interaction. This results in the amplified production of hydroxyl and superoxide free radicals through a Fenton–Haber/Weiss (respectively) reaction, which in turn leads to neurotoxic oxidative stress in cells that are in close proximity to Aβ plaques (Sayre et al., 1997). Numerous studies support the hypothesis that oxidative damage surrounding the Aβ plaques is involved in the neurodegenerative process of AD (Sayre et al., 1997; Lovell and Markesbery, 2007).

There are numerous contrasts between cortical Aβ plaques produced in AD and the APP/PS1 brains in terms of plaque morphology, iron management, and related inflammation (Table 2). Iron is frequently found in AD tissue samples, and is notably concentrated within the central core, diffusely within corona of the AD plaques, and throughout the tissue in the form of focal hemosiderin or magnetite iron deposits as a result of the breakdown of ferritin (Collingwood et al., 2008). In contrast, the cortical plaques observed in the transgenic mouse tissue contain trace amounts of diffuse iron; visibly less staining than surrounding cortical cells (Figure 5).

**Table 2:** **Summary of histological observations across tissue type**.

**Stain type**	**Human–Alzheimer's**	**Mouse–APP/PS1**
Iron	High association of iron with plaques, diffuse staining throughout tissue	Minimal staining, minute association with plaques
Aβ_40_	Core and coronal regions, close overlap with fibrillar thioflavin-S. Staining of cells outside plaques	Highly positive, exhibit protofibril staining beyond Thio-S boundary. Some staining of cells outside of plaques
Aβ_42_	Highly positive core, moderate staining in corona	No positive staining
Plaque Morphology	Small dense fibrillar core surrounded by diffuse coronal halo region	Globular, large dense fibrillar core, small coronal region
Astrocyte	High incidence of activated astrocytes around plaques	Random distribution of resting—lack of association with plaques
Microglia	Active microglia; close association around and inside of plaques	Modest active and ramified, association with plaque periphery
L-Ferritin	Throughout tissue and within coronal region of plaques—microglial morphology with some oligodendrocyte	Found throughout. Not associated with plaques and oligodendrocyte morphology
H-Ferritin	Highly positive in plaque core and cells throughout cortex with random distribution—neuronal in morphology	Minor positive staining in plaque core
Fluoro-jade C	Positive staining in cells that express Aβ_40_. Some association surrounding plaques	Central plaque core, no observable staining in plaque periphery

Alzheimer's Aβ plaques in this study exhibit a tight association with cells containing L-ferritin, congruent with previous literature (Lopes et al., 2008). The cellular morphology of L-ferritin positive cells is consistent with microglial IHC staining; indicating that microglial cells surrounding and infiltrating the plaques contain L-ferritin. Microglial and oligodendrocyte cells are both known to stain positively for L-ferritin (Connor et al., 1992b) and are differentiated from one another based on previously determined signature morphology (Lopes et al., 2008). Ferritin positive microglia exhibit fine cytoplasmic ramifications and irregularly-shaped elongated nuclei. Conversely, oligodendrocytes exhibit strong parinuclear staining with few processes and a large nucleus (Jellinger et al., 1990; Connor et al., 1992b; Lopes et al., 2008). Furthermore, previous work has utilized L-ferritin staining as a cellular marker for microglial cells (Kaneko et al., 1989) and ferritin accumulation around neuritic plaques is almost exclusively associated with microglial cells (Grundke-Iqbal et al., 1990). Microglial cells are known to harbor high quantities of the light ferritin isoforms for long-term iron storage, understood to be due to phagocytosis of apoptotic cells undergoing iron induced free-radical oxidative stress (Connor et al., 1994; Han et al., 2002; Mehlhase et al., 2006). In the APP/PS1 model, L-ferritin staining is found throughout the tissue samples within the granular cell layer and appears to be of oligodendrocyte origin based on cellular morphology (Figures 1, 3, comparatively). Having no light ferritin isoform associated with microglial cells in the APP/PS1 model is indicative of a system that is not undergoing excessive iron build-up. This finding is unlike the AD tissue where microglial cells are responding to cell-mediated inflammatory signaling and sequestering high amounts of iron during the phagocytic process.

H-ferritin neuronal staining observed within AD tissue is not detected to the same extent as within the transgenic mouse tissue. H-ferritin rapidly sequesters iron and is involved in excessive iron detoxification, thereby protecting against iron-induced oxidative damage (Connor et al., 1994; Telfer and Brock, 2002). Up-regulation of H- and L-ferritin transcription is known to occur in relation to increased iron presence, hypoxic incidents, as well as during enhanced inflammatory response (Rogers et al., 2008); all of which are involved in the AD process. The reduced inflammatory response and ferric iron concentration found in the APP/PS1 neural tissue, compared to the AD tissue, is consistent with the reduction of H-ferritin staining. The data are congruent with an overabundance of iron in the AD tissue and less global iron present in the APP/PS1 neural tissue.

Involvement of microglia and astrocytes is an important component in the pathogenesis of AD as these cells respond to neuronal environmental changes and produce numerous regulatory proteins, inflammatory cytokines, and protease inhibitors associated with inflammatory function (von Bernhardi and Ramirez, 2001). Although generally considered a positive presence during inflammatory response, there is growing support that microglia play a detrimental role in the AD process (Wegiel et al., 2000; von Bernhardi and Ramirez, 2001). The recruitment and activation of microglial cells in and around Aβ plaques can lead to the production of various cytokines and neurotoxins that are known to cause neuronal injury and death (Benveniste et al., 2001). A marked association is present between activated microglial cells and Aβ plaques in the AD samples. Microglial cells are seen surrounding and infiltrating the outer halo region in close proximity to the dense core of the neuritic AD plaques. Staining of the APP/PS1 tissue demonstrates an association between microglial cells and amyloid plaques, however reduction in ferritin staining within these microglia and lack of iron staining within APP/PS1 plaques would indicate that they are responding to the amyloid masses themselves, rather than increased iron deposition.

The AD tissue samples exhibit a pronounced astrocytic response with a clear relationship between plaque and astrocyte location. Well defined astrocyte somas are found along the periphery of the plaques with processes extending into the coronal halo region. The APP/PS1 samples demonstrate strong astrocyte staining throughout the tissue with minimal association to Aβ plaques. Previous study has revealed increased astrocytosis via cortical GFAP protein and mRNA measurement in whole brain homogenates of the transgenic APP/PS1 mice (Gordon et al., 2002; Gallagher et al., 2012). While the histological data conceivably support increased astrocytosis; the whole brain homogenates do not reflect the lack of an astrocyte and plaque association as seen regionally on histological images of the APP/PS1 tissue. Astrocytes are involved in the inflammatory response found in regions of neural-cellular distress and neurodegeneration; aiding in the distribution of metabolites to facilitate the repair of affected regions. Astrocytes in the AD tissue show a reduced star-like state with a large soma and prominent processes, which is typical of an astrocyte in response to cellular injury (Pekny and Nilsson, 2005; Schubert et al., 2009), potentially induced by neurotoxic accumulation of Aβ. In the transgenic model, the samples do not demonstrate the same response and are viewed as resting astrocytes in a metabolic helper state. It is of considerable interest that the APP/PS1 tissue does not demonstrate an astrocyte and plaque proximal relationship, yet exhibit microglia in close proximity with the periphery of the Aβ plaques. Microglia in both the AD and APP/PS1 tissue appear to be responding to the foreign nature of the amyloid plaque masses in an attempt to scavenge fibrillar beta-amyloid through phagocytosis (Koenigsknecht and Landreth, 2004). The negative astrocytic response to Aβ plaques in the APP/PS1 tissue is evidence of the lack of cellular degradation surrounding these plaques. This observation is furthered strengthened by the APP/PS1 FJC staining showing a decreased amount of degenerating neurons surrounding plaques. We hypothesize that astrocytes in the AD tissue are responding to both Aβ and cellular oxidative distress related to focal iron deposition. In addition, FJC staining of AD tissue shows a clear overlap in neuronal degeneration staining with Aβ. It is not clear from these stains if the cellular Aβ stained in the tissue samples is intra- or extra-cellular in origin or the solubility of the Aβ, both of which are associated with neuronal apoptosis (Kienlen-Campard et al., 2002).

The co-occurrence of iron mismanagement and natural production of aberrant Aβ fibrils in the AD brain are factors that appear to aid in plaque formation in a yet to be determined fashion. These factors are both markedly different in APP/PS1 neural tissue and it is plausible that the difference in amyloid fibril production and ferric iron concentrations found in the transgenic neural tissue partially account for the dissimilarity between AD and transgenic tissue. Iron aids in Aβ fibrillogenesis *in vitro* (Ryu et al., 2008) and it is hypothesized to play a role in *in vivo* plaque generation. Increased amounts of iron present in the AD brain along with fibrillogenic Aβ_42_ creates an environment suited for plaque formation. It has also been proposed that peptide sequences produced during APP cleavage act as a synergistic neuronal iron mitigation system. This hypothesis is supported by data showing that both APP and subsequent α-secretase cleavage is modulated by iron levels (Bodovitz et al., 1995; Avramovich-Tirosh et al., 2008; Carlson et al., 2008) and that APP is involved in cellular ferroportin iron export (Duce et al., 2010; Ayton et al., 2014; Wong et al., 2014). The APP/PS1 brain stages a neural environment upon which Aβ plaques form that is dissimilar to the human AD brain. Our data provide evidence of differential APP/PS1 plaque amyloid composition and iron load compared to those found in AD tissue. Transgenic plaques stained negatively for Aβ_42_ which is supported by the originators of this APP/PS1 mouse line (Borchelt et al., 1997) who have also shown reduced Aβ_42_ immunoreactivity. There also remains the possibility that modifications to the C-terminus end of Aβ_42_ in the APP/PS1 model are present, which can affect plaque morphology, iron-binding ability, and inhibit Aβ_42_ antibody binding.

While the relation of iron to native APP processing, Aβ generation, and amyloidogenesis is palpable, a clear causative pathway has not been established. The seeding and growth of beta-amyloid plaques in the human brain is influenced by a myriad of factors, not limited to the influence of transition metals (iron, copper, aluminum, and zinc), genetics, and protein thermodynamics, all of which influence the multifaceted AD process. As a concomitant loss of iron control is not occurring in mouse brain tissue, the expression and processing of endogenous APP is occurring differently. Increased production of human APP and PS1 in the APP/PS1 model is transgenetically modulated and transcription is controlled by the native mouse prion protein promoter. As such, the time course and evolution of AD and APP/PS1 Aβ plaques is inherently different, with APP/PS1 plaque progression being simplistic in comparison. In addition, the APP/PS1 mouse model does not exhibit phosphorylated tau (pTau). The literature bases provides indication of a relationship between iron and pTau pathology whereupon iron chelation impedes the formation of phosphorylated tau (Guo et al., 2013b); however, the role of pTau pathology in the low iron environment of the transgenic brain is unclear.

The present study outlines the association of iron content with beta-amyloid plaques and postulates the effect of iron on amyloidosis. With this imparted, the interpretations above should be considered in the context of the wider literature and how these relate to potential study confines. The literature base contains some heterogeneity in regard to the effect of formalin fixation on tissue iron content, specifically in brain tissue. The data in the current study demonstrates that formalin fixed human AD tissue exhibits more iron compared to APP/PS1 brain tissue, even with extended fixation of the human tissue. The high binding affinity of beta-amyloid for iron (Jiang et al., 2009) suggests that iron bound to the plaques remains largely unaffected and that unbound iron makes the bulk of any leached iron. Additionally, mouse regions known to have high iron content, such as the substantia nigra, stained highly positive for iron (not shown). Furthermore, the visual interpretation of the histological results is qualitative in nature and care must be taken when comparing these results to quantitative metrics. Translation of the transgenic animal age to Alzheimer's tissue presents a challenge, especially in regard to late-stage plaque progression. Evaluating aged APP/PS1 animals extends the opportunity to study iron related plaque interactions in late-stage plaques, similar to terminal AD patient tissue.

In summary, the data in this report provide evidence of numerous contrasts between AD and the APP/PS1 transgenic model aimed at mimicking the genesis of Aβ plaque formation. It is largely unknown as to why human AD neuronal tissue produces Aβ plaques, as the increase in the amyloidogenic Aβ peptide pathway is multifaceted and represents a misregulation of numerous endogenous systems. The current state of the amyloid cascade hypothesis regards beta-amyloid as one of many factors of the disease process (Pimplikar, 2009). For the most part, the same complexity does not hold true for the transgenic animal tissue for which Aβ and plaque formation is governed by the increased production of two introduced human mutations. The divergence between AD and APP/PS1 in plaque iron, morphology, and inflammatory response suggest that the transgenic model loosely fits within the current framework of the amyloid cascade model (Hardy and Allsop, 1991). As such, the differences between the plaques in the transgenic and AD tissue sample are of considerable importance to researchers whom are considering using the model for comparative Alzheimer's studies. The plaque deviations in the APP/PS1 model should be taken into account when translating pharmacological or antibody methodologies, such as iron chelation to mediate toxicity (Guo et al., 2013a), the augmentation of the immune system's inflammatory response (Mengel et al., 2013), the alteration of gamma and/or beta secretase mediated Aβ production (Hajos et al., 2013), or clearance of the amyloid-β peptide sequence (Malm et al., 2012). When interpreting results from the transgenic model and translating these to human AD trials, care must be taken to acknowledge the caveat that there is considerable divergence between the APP/PS1 transgenic model and AD with regard to iron concentration, inflammatory response, and structural morphology associated with Aβ plaques.

### Conflict of interest statement

The authors declare that the research was conducted in the absence of any commercial or financial relationships that could be construed as a potential conflict of interest.
